# Photodynamic Inactivation of *Candida albicans* in Blood Plasma and Whole Blood

**DOI:** 10.3390/antibiotics8040221

**Published:** 2019-11-13

**Authors:** Vera Sousa, Ana T. P. C. Gomes, Américo Freitas, Maria A. F. Faustino, Maria G. P. M. S. Neves, Adelaide Almeida

**Affiliations:** 1 Department of Biology & CESAM, University of Aveiro, Aveiro 3810-193, Portugal; vcfs@live.ua.pt; 2 Clinical Analysis Laboratory Avelab, Rua Cerâmica do Vouga, Aveiro 3800-011, Portugal; geral@avelab.pt; 3 Department of Chemistry & QOPNA and LAQV-REQUIMTE, University of Aveiro, Aveiro 3810-193, Portugal; gneves@ua.pt

**Keywords:** Antimicrobial Photodynamic Therapy, *Candida albicans*, porphyrins, methylene blue, blood plasma

## Abstract

The few approved disinfection techniques for blood derivatives promote damage in the blood components, representing risks for the transfusion receptor. Antimicrobial photodynamic therapy (aPDT) seems to be a promising approach for the photoinactivation of pathogens in blood, but only three photosensitizers (PSs) have been approved, methylene blue (MB) for plasma and riboflavin and amotosalen for plasma and platelets. In this study, the efficiency of the porphyrinic photosensitizer Tri-Py(+)-Me and of the porphyrinic formulation FORM was studied in the photoinactivation of *Candida albicans* in plasma and in whole blood and the results were compared to the ones obtained with the already approved PS MB. The results show that FORM and Tri-Py(+)-Me are promising PSs to inactivate *C. albicans* in plasma. Although in whole blood the inactivation rates obtained were higher than the ones obtained with MB, further improvements are required. None of these PSs had promoted hemolysis at the isotonic conditions when hemolysis was evaluated in whole blood and after the addition of treated plasma with these PSs to concentrates of red blood cells.

## 1. Introduction

Human blood is a key tissue responsible for transporting vital molecules like oxygen, carbon dioxide and glucose to the different parts of the body. This circulating fluid is composed of plasma, erythrocytes or red blood cells, leukocytes or white blood cells and thrombocytes or platelets [1,2,3]. Plasma constitute about 55% of whole blood, the red blood cells about 45% and white blood cells less than 1% [3].

Blood transfusions are usually required by patients with hemorrhagic diseases, anemia and after major surgeries, etc. This medical procedure can occur by whole blood transfusion and more commonly, by transfusions of blood components such as erythrocytes, plasma or platelets. In some cases, multiple blood transfusions are required to meet the body’s needs. Transfusions of erythrocytes concentrates are used in hypoxia conditions by blood loss after trauma or surgery [4,5,6]. These erythrocytes concentrates can be stored for 35 days at 2–6 °C in red cell preservation solutions [5,7]. Platelet transfusions are used for preventing or treating bleeding in patients with thrombocytopenia or abnormalities of platelet function [8,9]. It can be concentrated from plasma or by plateletpheresis from a single donor and stored at 22 °C for 5 days [10]. Plasma transfusions are required to correct deficiencies of clotting factors, for which a specific concentrate is not available, in patients in severe bleeding [4]. Plasma can be frozen promptly, stored at −18 °C for 5 years and could be defrosted before use.

To ensure the safety of transfusions in the blood collection, several procedures are adopted, including donor screening, specific serological and nucleic acid testing and transfusion hemovigilance. Despite the measures already adopted, microbial infections are yet transmitted through blood products transfusion [5,11,12] causing diseases in the blood receptor [5,13,14]. In the US, bacterial contamination is considered the second most common reason of death from a transfusion, resulting in morbidity and mortality from 100 to 150 transfused individuals each year [15]. Between 2010 and 2013, 111 transfusion-transmitted infections (TTIs) were detected in the European Union being 66% bacterial, 32% viral and less than 3% parasites [16,17]. Due to the combination of plasma fractions collected from different donors, the infections rates are more noteworthy in transfusions involving blood plasma or products derived from plasma [5,7]. It is in this context that the disinfection of blood and blood products assume great importance.

The conventional treatments used to inactivate pathogenic microorganisms in blood and blood products were developed essentially to inactivate virus [5,7,18]. The World Health Organization (WHO) recommends the screening for the presence of Human Immunodeficiency Virus (HIV), Hepatitis B Virus (HBV), Hepatitis C Virus (HCV) and the bacterium *Treponema pallidum* (causative agent of syphilis), for all blood donations [19]. However, the collected blood can contain other pathogenic agents like fungi [20], parasites [21,22] and several bacteria [5,15,23,24], which come especially from the intestine or from pre-symptomatic infections or coming from exogenous microorganisms, such as from skin. Nowadays, there are some available methods for the reduction of pathogens in blood products, but they are approved essentially for plasma.

The most generalized method combines the use of tri(*n*-butyl)phosphate and detergent Tween 80 and can be only used in plasma or protein concentrates. Due to the negative effects that these chemicals promote in the erythrocyte membranes and platelets, they must be removed after treatment [7,25,26,27]. The use of ultraviolet (UV) light is also considered and this methodology is essentially applied in plasma and platelet disinfection. The irradiation causes damage to the microbial genetic material avoiding its replication; however, this technique produces free radicals that are extremely cytotoxic [5,7,28,29]. Other processes used in blood purification such as chromatographic techniques using specific antibodies adsorbed and even physical methods to remove extracellular pathogens, like nanofiltration or cell washing are also in use for plasma [5,7,30]. However, they cannot be applied in concentrated platelets and erythrocytes since cell membranes can bind non-specifically to the antibodies and intracellular pathogens are not filtrated or wash removed by these techniques [5,7,30].

Another approach for blood disinfection is the antimicrobial photodynamic therapy (aPDT). In this therapeutic approach the combination of a photosensitizer (PS) and visible light in the presence of molecular oxygen, produces highly toxic oxygen species (ROS), such as singlet oxygen (^1^O_2_), hydrogen peroxide (H_2_O_2_), superoxide (O_2_^•−^) and hydroxyl radicals (OH^•^) [31,32]. These species are responsible for the irreversible oxidation of vital constituents of microorganisms, causing their death [32,33]. Nowadays, only three Ps [amotosalen (a psolaren), riboflavin (or vitamin B2) and methylene blue (MB)] are approved for blood disinfection and these can only be used for plasma and platelets treatment. The aPDT with amotosalen and riboflavin requires the use of UV (UVA) and UV (UVB), respectively, which may lead to the formation of harmful free radicals. Despite the radical species formed affect plasma proteins and platelets, the amotosalen and riboflavin were approved in Europe for platelets and plasma disinfection [12,34,35]. Contrarily, the aPDT with MB uses visible light instead of UV light and it is also approved for pathogen inactivation in plasma units [7,36]. In this case most enveloped viruses are inactivated, but non-enveloped viruses, intracellular viruses, protozoa, fungi and bacteria remain unaffected [7,37]. Due to the poor intracellular uptake of MB, this photosensitizer cannot inactivate intracellular pathogens [5,12,38,39]. Although MB may interact with clotting factors and most noticeably fibrinogen and factor VIII with a loss of approximately 30%, no relevant side effects were detected in patients transfused with MB-treated plasma [12,40,41]. Nevertheless, some doubts have arisen about the efficacy of MB in the treatment of plasma when used as a replacement solution for plasma exchange in the treatment of patients suffering from thrombotic thrombocytopenic purpura [36,42]. Although it is approved for the disinfection of plasma in several European countries, in France it was removed from the market due to allergic reactions detected in a few patients that received plasma treated with MB [36].

In what concerns to the disinfection of whole blood and erythrocytes concentrates, there is a great difficulty in the development of effective methodologies due to the complexity and sensitivity of the matrix. A recent study shows that the use of S-303, a positively charged synthetic alkylating agent, can disrupt the pathogen genetic material in erythrocytes [12,13,43,44]. Besides this promising study, no well-established method for whole blood disinfection was approved.

Having in mind the lack of more efficient methods for the inactivation of pathogens in blood products, the combination of MB as a PS and visible light based in the photodynamic therapy effect seems to be the more consensual method for the pathogenic inactivation in plasma. In fact, aPDT represents a non-antibiotic approach, that has been shown to be effective in the photoinactivation of bacteria, viruses, fungi, and protozoa [45]. aPDT action is multi-target, with the great advantage over traditional approaches, which means that this therapy acts on a variety of biochemical targets (extra and intracellular structures) and therefore prevents the development of microbial resistance mechanisms [46]. In fact, repeated photosensitization does not induce resistance in microorganisms [33,45]. However, fungi are more complex microorganisms and therefore become more challenging targets than viruses and bacteria. Fungi inactivation seems to be less dependent on the binding of PS to cells. In the case of fungi, the free PS induces some initial changes in the plasmatic membrane and then penetrate in the cell, causing more extensive damage in more complex subcellular structures such as mitochondria or nucleus [45,47].

There are few studies reporting fungal infections transmitted by blood transfusions. In 2011, a statistical study analyzed 86 patients with severe abdominal sepsis and severe pancreatitis, in which 23% of these patients were colonized by *Candida* and 8% of the patients developed candidiasis after transfusion of at least four volumes of red blood cells [48]. *C. albicans* is a commensal fungus that can be isolated from the gastrointestinal tract, oral and vaginal mucosa of healthy individuals, existing in balance with the bacterial flora and host immune system [49]. This fungus presents high ability to survive and proliferate in adverse environments with drastic changes in oxygen, carbon dioxide, nutrients, pH, osmolality and temperature [47]. Another important feature of *C. albicans* is its ability to form biofilms, that are a problem in medical practice because they can be formed in artificial heart valves and dentures, presenting resistance to various antifungal agents currently used in clinical practice, including amphotericin B and fluconazole, and have multiple mechanisms of resistance [50]. In disseminated candidiasis in individuals with the compromised immune system, *C. albicans* gains direct access to the bloodstream with a mortality rate of approximately 40% [51,52].

*C. albicans* has been shown to be susceptible to aPDT in their planktonic [53,54] and biofilms forms [54,55]. Moreover, this therapy appears to prevent the formation of biofilms by reducing their adhesion capacity [56]. The haematoporphyrin [57], Photofrin [58,59], *meso*-tetrakis(*N*-methyl-6-quinolinyl)-substituted porphyrins and chlorins [60], cationic derivatives of *meso*-tetrapyridylporphyrin [61], porphyrin derivatives bearing a fluconazole unit [62] and ALA [63] are some of the PS used in vitro assays that efficiently inactivate *C. albicans*.

**Figure 1:**
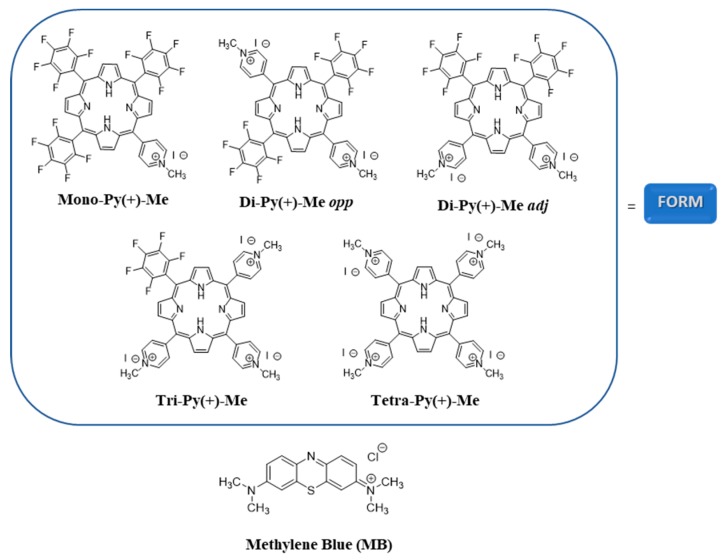
Structures of the photosensitizers (PSs) used in this study to photoinactivate *C. albicans*.

In this work, we report the study of the photodynamic effect of 5,10,-15-tris(1-methylpyridinium-4-yl)-20-(pentafluorophenyl)porphyrin tri-iodide (Tri-Py(+)-Me) and a PS formulation (FORM), based on a non-separated mixture of five cationic *meso*-tetraarylporphyrins (Figure 1), in the photoinactivation of *C. albicans* in blood plasma and in whole blood. The way that concentrates of red blood cells are affected by the PS concentration and by plasma after being submitted to aPDT was also evaluated. All the results were compared with the ones achieved with the approved MB. The selected porphyrinic PSs have already proved their efficiency in the photoinactivation of *Escherichia coli* [5,64,65], *Pseudomonas syringe* [66], *Staphylococcus aureus* [5,64,65], and *C. albicans* [54]. FORM has been considered an excellent alternative to the highly efficient constituents, Tri-Py(+)-Me and Tetra-Py(+)-Me, since production costs were reduced significantly due to its use [54,64,65]. Moreover, Tri-Py(+)-Me was recently described as a potential PS for the inactivation of *E. coli* and *S. aureus* in blood plasma and whole blood [5]. In this report, this PS had promoted the total inactivation of *S. aureus* in blood plasma and *c.a.* of 4 log_10_ in *S. aureus* viability in whole blood. In the case of *E. coli*, a reduction in the survival of this bacterium of *c.a*. 6 log_10_ and 5 log_10_ was achieved for plasma and whole blood, respectively. Furthermore, it was demonstrated that the Tri-Py(+)-Me does not promote osmotic stress [5].

## 2. Results

### 2.1. Photodynamic Efficiency of FORM, Tri-Py(+)-Me, Tetra-Py(+)-Me, and MB in the Inactivation of C. albicans in PBS

**Figure 2:**
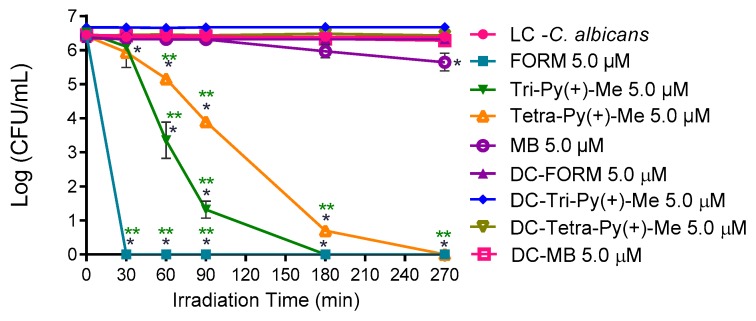
Photodynamic inactivation of *C. albicans* in the presence of FORM, Tri-Py(+)-Me, Tetra-Py(+)-Me, and MB at 5.0 μM in PBS and irradiated with white light (380–700 nm) at 2.5 mW/cm^2^. Values represent the average of three independent experiments with two replicates each; error bars indicate the standard deviation. Lines just combine the experimental points. * *p* < 0.05 (relatively to the LC); ** *p* < 0.05 (relatively to MB).

Figure 2 presents the photodynamic inactivation profile of *C. albicans* in PBS in the presence of FORM, Tri-Py(+)-Me, Tetra-Py(+)-Me, and MB at 5.0 μM when irradiated with white light (380–700 nm) at 2.5 mW·cm^−2^. The results showed that the porphyrin derivatives are effective in the photodynamic inactivation of *C. albicans*, promoting a decrease in the survival of the fungus until the detection limit was reached after 30, 180, and 270 min of irradiation, for FORM, Tri-Py(+)-Me, and Tetra-Py(+)-Me, respectively. It is noteworthy that FORM achieved the highest photoinactivation rate (a decrease of 0.6 log_10_; ANOVA, *p* < 0.05) of *C. albicans* in the shortest light exposure time. The MB (used as PS reference), was less effective, causing a decrease of 0.8 log_10_ in *C. calbicans* survival after 270 min of irradiation.

### 2.2. Evaluation of aPDT Effect on Erythrocyte Osmotic Fragility

In order to choose a safe concentration of each PS to be used in the aPDT of *C. albicans* in blood plasma and whole blood, the erythrocyte osmotic fragility was assessed at different concentrations of each PS (5.0, 10, and 20 μM) before (0 min) and after aPDT treatment (90 min). Concentrations that did not promote hemolysis after the aPDT protocol were used in the photoinactivation of *C. albicans* in plasma and in whole blood. Since Tetra-Py(+)-Me was the least efficient porphyrinic PS in the *C. albicans* photoinactivation in PBS, this PS was not included in the following studies.

Thus, blood samples before and after exposure to aPDT protocol (incubation with each PS concentration followed by irradiation with white light at 150 mW·cm^−2^ for 90 min) were added to tubes containing increasing concentrations of sodium chloride (NaCl) solution (0, 0.1, 0.3, 0.5, 0.7, and 0.9%) at pH 7.4 and the hemoglobin was spectrophotometrically quantified. The results of the erythrocyte osmotic fragility of Tri-Py(+)-Me were already reported and had shown that this PS did not promote significant (ANOVA, *p* > 0.05) erythrocytes hemolysis after aPDT at 5.0 and 10 μM using a non-stress (isotonic) condition (0.9% NaCl) [5].

**Figure 3:**
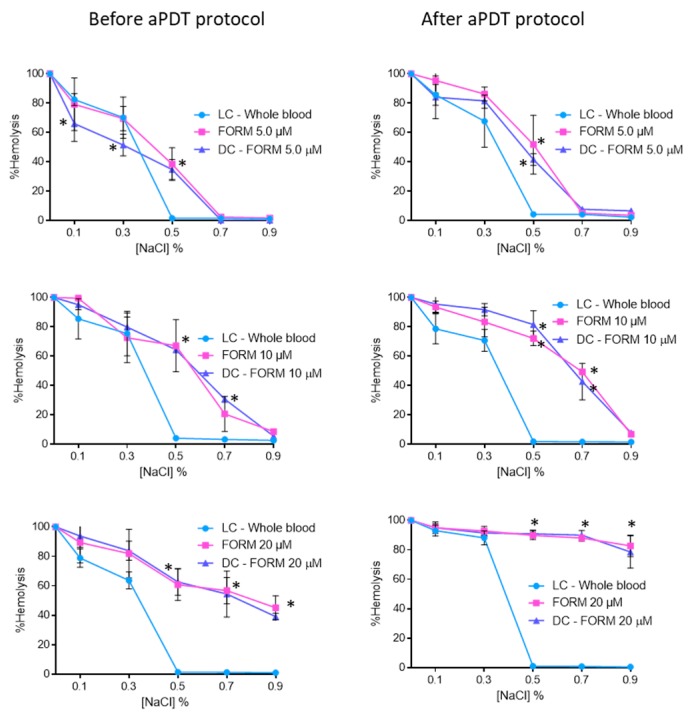
Erythrocyte osmotic fragility before and after aPDT treatment under white light (380–700 nm) at an irradiance of 150 mW·cm^−2^, with FORM at 5.0, 10, and 20 μM. Light control (erythrocytes under light) and dark control (erythrocytes incubated with FORM without irradiation) were included. Values represent the average of three independent experiments; error bars indicate the standard deviation. Lines just combine the experimental points. * *p* < 0.05 (relatively to the LC).

Figure 3 shows the results achieved for osmotic erythrocyte fragility promoted by FORM at 5.0, 10, and 20 μM before and after the aPDT protocol. In the isotonic solution (0.9% NaCl) and for the concentration of 5.0 μM, no significant (ANOVA, *p* > 0.05) erythrocytes hemolysis were observed. The same profile was attained for the NaCl solution at 0.7%. However, when submitted to a 0.5% NaCl solution, FORM at 5.0 μM endorsed hemolysis rates of 38% (*p* < 0.05) and 51% (ANOVA, *p* < 0.05) before and after aPDT, respectively. In this case, no significant differences were observed between dark control and irradiated samples treated with FORM. Also, for FORM at 10 μM, observed conditions were not considered significant (ANOVA, *p* > 0.05) for erythrocytes hemolysis under non-stress (isotonic) conditions, both before and after aPDT protocol. However, for the NaCl solution at 0.7%, hemolysis rates of 20% (ANOVA, *p* < 0.05) and 42% (ANOVA, *p* < 0.05) were observed before and after irradiation. For the NaCl solutions at 0.5% and at lower concentrations, the hemolysis observed increases (higher than 64%), which was significantly different from the hemolysis rate achieved in the light control (ANOVA, *p* < 0.05). Also in this case, no significant differences were observed between dark control and irradiated samples treated with FORM. When the osmotic erythrocyte fragility was studied with FORM at 20 μM, high hemolysis rates were observed in all NaCl solutions, even before the aPDT protocol. For example, in the isotonic solution (0.9% NaCl), 45% (ANOVA, *p* < 0.05) and 82% (ANOVA, *p* < 0.05) of hemolysis was observed, before and after aPDT, respectively. In fact, after irradiation, in all the NaCl solutions the observed hemolysis was higher than 80%. Once again, no significant differences were observed between dark control and irradiated samples treated with FORM.

**Figure 4:**
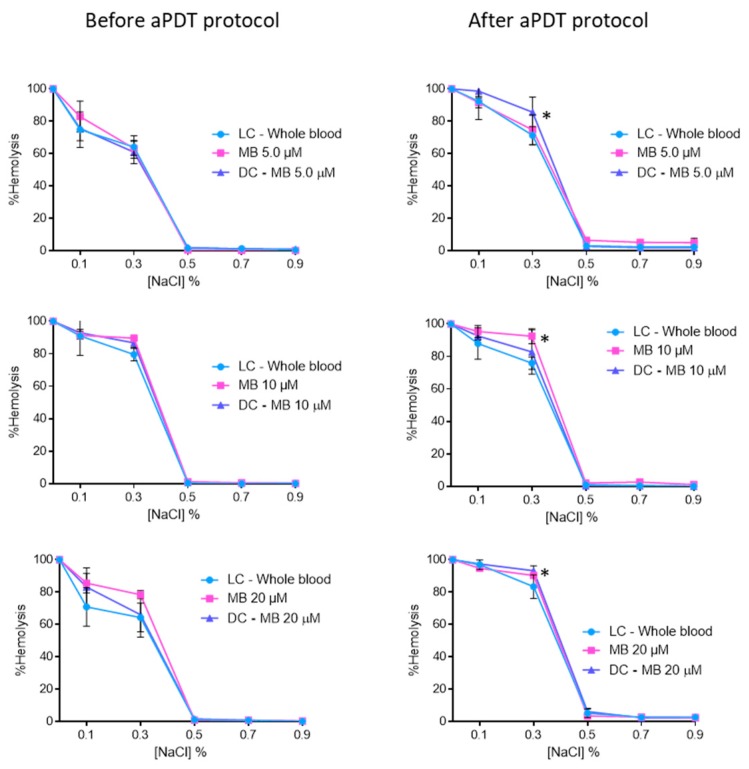
Erythrocyte osmotic fragility before and after aPDT treatment under white light (380–700 nm) at an irradiance of 150 mW·cm^−2^, with MB at 5.0, 10, and 20 μM. Light control (erythrocytes under light) and dark control (erythrocytes incubated with MB without irradiation) were included. Values represent the average of three independent experiments; error bars indicate the standard deviation. Lines just combine the experimental points. * *p* < 0.05 (relatively to the LC).

The results of the osmotic erythrocyte fragility tests with MB at 5.0, 10, and 20 μM are presented in Figure 4. In all solutions with percentages of NaCl equal to 0.5% or higher, no significant hemolysis was observed. For MB at 5.0 μM, 10, and 20 μM after the irradiation protocol, it was possible to observe hemolysis rates of 71% (ANOVA, *p* < 0.05), 92% (ANOVA, *p* < 0.05), and 93% (ANOVA, *p* < 0.05) for the 0.3% NaCl solution, respectively. In all cases no significant differences were observed between dark control and irradiated MB.

**Figure 5:**
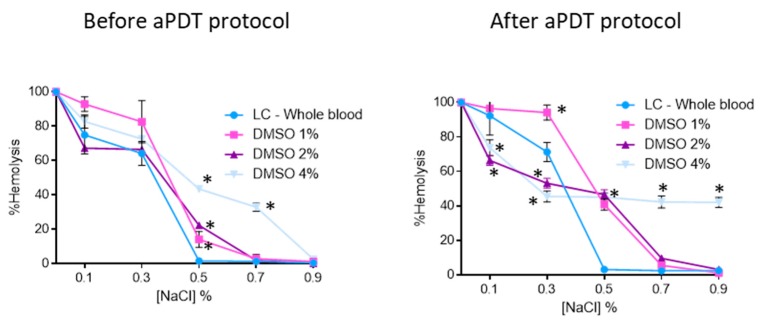
Erythrocyte osmotic fragility before and after aPDT treatment under white light (380–700 nm) at an irradiance of 150 mW·cm^−2^, with 1%, 2%, and 4% of DMSO. Light control (erythrocytes under light) was included. Values represent the average of three independent experiments; error bars indicate the standard deviation. Lines just combine the experimental points. * *p* < 0.05 (relatively to the LC).

Contrary to the MB stock solution, which was prepared in PBS, the porphyrinic stock solutions were prepared in DMSO (FORM and Tri-Py(+)-Me) due to their limited solubility in aqueous solutions. So, the osmotic erythrocyte fragility promoted by DMSO was accessed in the same percentages used in the assays with each porphyrinic PS. Thus, 1%, 2% and 4% of DMSO were added to blood samples and submitted to the aPDT protocol (irradiation with white light at 150 mW·cm^−2^ for 90 min). The samples were added to tubes containing increasing concentration of NaCl solution (0%, 0.1%, 0.3%, 0.5%, 0.7%, and 0.9%) at pH 7.4 and the hemoglobin was quantified before and after the irradiation procedure. The results obtained are presented in Figure 5. As it is possible to observe, samples with 1% and 2% of DMSO did not promote significant hemolysis in the 0.7% and 0.9% NaCl solutions either before and after the aPDT protocol. However, in the solutions with higher percentages of NaCl the hemolysis rates promoted by these percentages of DMSO increased; before the irradiation, the erythrocyte solutions with 1% and 2% of DMSO suffered a hemolysis rate of 14 (ANOVA, *p* < 0.05) and 22% (ANOVA, *p* < 0.05), respectively, in the NaCl solution at 0.5%. After the aPDT protocol, these conditions promoted higher hemolysis: 40 (ANOVA, *p* < 0.05) and 45% (ANOVA, *p* < 0.05), for 1% and 2% of DMSO, respectively. When the osmotic erythrocyte fragility was studied with 4% of DMSO high hemolysis rates were observed in all NaCl solutions, even before the irradiation protocol, with the exception of the isotonic solution before the aPDT protocol, where no hemolysis was observed. After the irradiation procedure and in NaCl solutions at 0.3%, 0.5%, 0.7%, and 0.9%, the hemolysis rates were 45% (ANOVA, *p* < 0.05).

### 2.3. aPDT of C. albicans in Blood Plasma using FORM, Tri-Py(+)-Me, and MB

**Figure 6:**
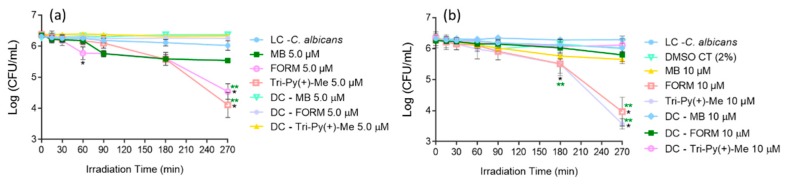
Photodynamic inactivation of *C. albicans* in the presence of FORM, Tri-Py(+)-Me, and MB at 5.0 (**a**) and 10 μM (**b**) in blood plasma and irradiated with white light at at 150 mW·cm^−2^. Values represent the average of three independent experiments; error bars indicate the standard deviation. Lines just combine the experimental points. * *p* < 0.05 (relatively to the LC); ** *p* < 0.05 (relatively to MB).

After the erythrocyte osmotic fragility studies, the PS concentrations that did not promote significant hemolysis in the isotonic conditions were used in the inactivation of *C. albicans* in blood plasma. Thus, FORM, Tri-Py(+)-Me, and MB at 5.0 and 10 μM were tested against the fungus strain in plasma, applying the same irradiation conditions used in the erythrocyte osmotic fragility studies (white light at 150 mW·cm^−2^) and the results are presented in Figure 6.

The results showed that FORM at 5.0 (Figure 6a) and 10 μM (Figure 6b) was capable of photoinactivating *C. albicans* in blood plasma. When compared with the light control (LC), FORM promoted a decrease of 1.2 log_10_ (ANOVA, *p* < 0.05) at 5.0 μM and 1.7 log_10_ (ANOVA, *p* < 0.05) at 10 μM in the fungus survival rate after 270 min of light irradiation. No effects on C. albicans survival rates were observed in dark controls (DC) as well in the DMSO control (DMSO CT).

When Tri-Py(+)-Me was used as PS in the photoinactivation of *C. albicans* in blood plasma (Figure 6), the photodynamic profile attained was similar to the one observed for FORM, although the decrease in the fungus survival rate was significantly higher. As is possible to observe, Tri-Py(+)-Me at 10 μM and after 180 min of irradiation had promoted a decrease of 0.6 log_10_ (ANOVA, *p* < 0.05) when compared with LC. After 270 min of aPDT, a decrease of 1.9 and 2.5 log_10_ (ANOVA, *p* < 0.05) in the *C. albicans* survival was achieved at 5.0 and 10 μM of Tri-Py(+)-Me, respectively. As in the previous case, no significant effects in the *C. albicans* survival were observed in dark controls (DC).

The reference PS MB was shown to be the least efficient PS in the photoinactivation of C. albicans in blood plasma, causing a tiny decrease in fungus survival: 0.5 and 0.4 log_10_ (ANOVA, *p* < 0.05), for 5.0 and 10 μM, respectively, after 180 min of aPDT (Figure 6). It is important to note that the photodynamic inactivation profile remained almost constant between 90 and 270 min of irradiation. It was also observed that the blue color of the plasma solution, which was present in the beginning of the aPDT protocol, disappeared throughout the photodynamic process. Also in this case, no significant effects in the C. albicans survival were observed in dark controls (DC).

### 2.4. aPDT of C. albicans in Whole Blood using FORM and Tri-Py-(+)-Me

**Figure 7:**
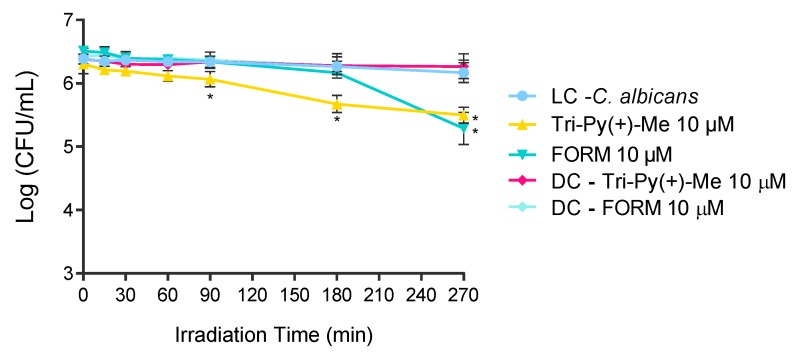
Photodynamic inactivation of *C. albicans* in the presence of FORM and Tri-Py-Me(+)-PF at 10 μM in whole blood and irradiated with white light at at 150 mW·cm^−2^. Values represent the average of three independent experiments; error bars indicate the standard deviation. Lines just combine the experimental points. * *p* < 0.05 (relatively to the LC).

The most promising PSs in the photoinactivation of *C. albicans* in blood plasma were used to photoinactivate this fungus in whole blood artificially contaminated FORM and Tri-Py(+)Me at 10 μM. The results presented in Figure 7, show that the photoinactivation of *C. albicans* in the presence of FORM started after 180 min of irradiation, causing a decrease of 0.7 log_10_ (ANOVA, *p* < 0.05) in its survival after 270 min of treatment. In the case of Tri-Py(+)-Me (Figure 7), the decrease in *C. albicans* survival began at 90 min of irradiation, and reached a decrease in the fungus survival of 0.6 log_10_ (ANOVA, *p* < 0.05) and 0.7 log_10_ (ANOVA, *p* < 0.05) after 180 min and 270 min of aPDT protocol, respectively. In both cases, no significant reduction on the *C. albicans* survival was achieved in the dark controls.

### 2.5. Evaluation of Erythrocyte Osmotic Fragility after the Addition of the Treated Plasma with FORM, Tri-Py(+)-Me and MB to the Concentrated Erythrocytes

Having in mind the potential application of FORM and Tri-Py(+)-Me as PSs in the *C. albicans* inactivation in blood plasma, the erythrocyte osmotic fragility after the addition of treated plasma to the concentrated erythrocytes was assessed. Thus, blood plasma solutions were submitted to aPDT protocol (described for the blood plasma photodynamic assays) in the presence of FORM, Tri-Py(+)-Me, and MB at 5.0 and 10 μM. After aPDT, aliquots of treated plasma were added to the concentrated erythrocytes and then osmotic fragility was assessed after 30 min and 6 h of incubation in the dark (see Figure 11).

**Figure 8:**
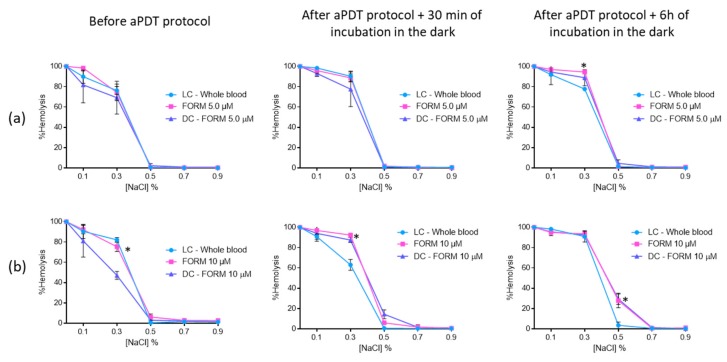
Erythrocyte osmotic fragility after the addition of the treated plasma with 5.0 (**a**) and 10 μM (**b**) of FORM to the concentrated erythrocytes before and after aPDT treatment under white light (380–700 nm) with an irradiance of 150 mW·cm^−2^. Light control (plasma under light+concentrated erythrocytes) and dark control (plasma incubated with FORM without irradiation+concentrated erythrocytes) were included. Values represent the average of three independent experiments; error bars indicate the standard deviation. Lines just combine the experimental points. * *p* < 0.05 (relatively to the LC).

The results had shown that plasma treated with FORM at 5.0 μM did not promoted significant erythrocyte (ANOVA, *p* > 0.05) hemolysis before aPDT procedure and after 30 min of incubation in the dark, since no significant differences were observed between LC and FORM, in NaCl solutions with percentages higher than 0.3% (Figure 8a). After 6 h of incubation in the dark, it was possible to observe 94% (ANOVA, *p* < 0.05) of hemolysis in 0.3% of NaCl solution in FORM, 17% higher than the hemolysis achieved in LC. However, in solutions with higher percentages of NaCl, no hemolysis was achieved. For FORM at 10 μM (Figure 8b), significant (ANOVA, *p* < 0.05) hemolysis was attained for 0.3% and 0.5% of NaCl solution before aPDT protocol (c.a 87% and 5.9%, respectively). However, after 6 h of incubation in the dark, 27% of hemolysis was only observed in 0.5% of NaCl solution. No significant differences (ANOVA, *p* > 0.05) were observed between dark control and irradiated FORM.

**Figure 9:**
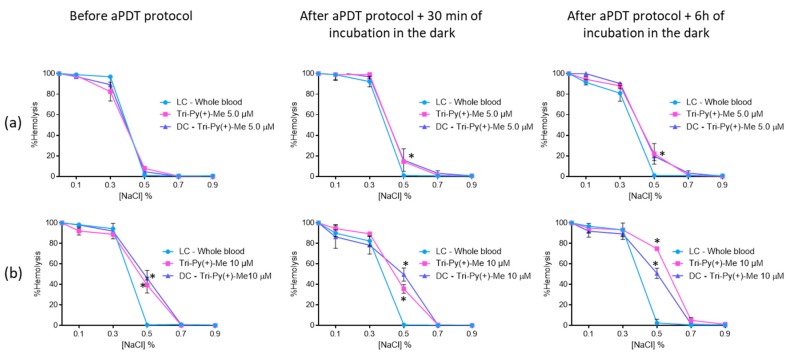
Erythrocyte osmotic fragility after the addition of the treated plasma with 5.0 (**a**) and 10 μM (**b**) of Tri-Py(+)-Me to the concentrated erythrocytes before and after aPDT treatment under white light (380–700 nm) with an irradiance of 150 mW·cm^−2^. Light control (plasma under light+concentrated erythrocytes) and dark control (plasma incubated with Tri-Py(+)-Me without irradiation+concentrated erythrocytes) were included. Values represent the average of three independent experiments; error bars indicate the standard deviation. Lines just combine the experimental points. * *p* < 0.05 (relatively to the LC).

When the concentrated erythrocytes was a mixture with plasma treated with Tri-Py(+)-Me at 5.0 μM (Figure 9a), it was observed that significant hemolysis in the 0.5% NaCl solution only occurred after the aPDT protocol: 15% after 30 min and 22% after 6 h of dark incubation. In the solutions with 0.7% and 0.9% of NaCl, no hemolysis was observed.

At the higher concentration (10 μM, Figure 9b) of Tri-Py(+)-Me, a similar profile was attained at 0.7% and 0.9% NaCl concentrations. For the 0.5% NaCl solution, significant hemolysis was observed before (39% (*p* < 0.05)) and after aPDT protocol, with dark incubations at 30 min of [35% (ANOVA, *p* < 0.0.5)] at 6 h (74% (ANOVA, *p* < 0.05)). Also in this case, no significant differences were observed between dark control and irradiated Tri-Py(+)-Me, with the exception of DC after 6 h of dark incubation. In this case, the hemolysis observed for irradiated Tri-Py(+)-Me was higher than the one observed for DC (75% vs 51% (ANOVA, *p* < 0.05)).

**Figure 10:**
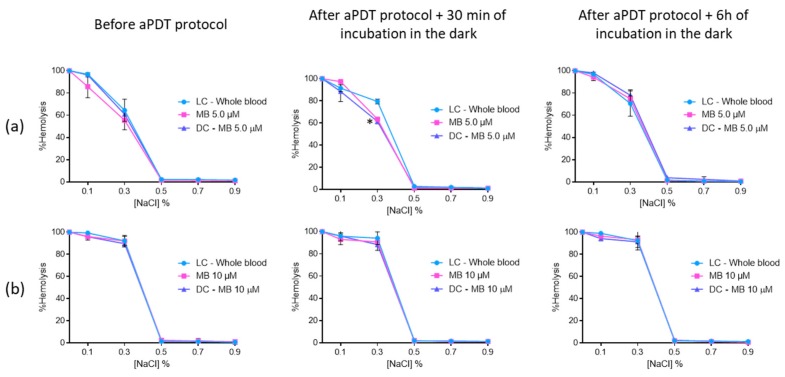
Erythrocyte osmotic fragility after the addition of the treated plasma with 5.0 (**a**) and 10 μM (**b**) of MB to the concentrated erythrocytes before and after aPDT treatment under white light (380–700 nm) with an irradiance of 150 mW·cm^−2^. Light control (plasma under light+concentrated erythrocytes) and dark control (plasma incubated with MB without irradiation+concentrated erythrocytes) were included. Values represent the average of three independent experiments; error bars indicate the standard deviation. Lines just combine the experimental points. * *p* < 0.05 (relatively to the LC).

The erythrocyte osmotic fragility results with MB revealed that this PS did not endorse hemolysis before and after aPDT for the two concentrations evaluated (Figure 10). The only exception observed was for 0.3% NaCl solution, where the hemolysis of MB at 5.0 μM [65% (ANOVA, *p* < 0.05)] and the respective DC (75% (*p* < 0.05)) was lower than the one observed for the LC (83% (ANOVA, *p* < 0.05)). Nevertheless, this feature seems to be irrelevant after 6 h of incubation in the dark, since no differences were observed between LC, DC, and irradiated MB.

## 3. Discussion

*C. albicans* is a commensal microorganism colonizing the gastrointestinal tract, skin, oral cavity, and reproductive tract in an asymptomatic and healthy way, but, under specific conditions, may cause nosocomial infections through the bloodstream [67]. Our results demonstrated that *C. albicans* is susceptible to aPDT in PBS, confirming the data in the literature that shows several examples where *C. albicans* planktonic cells as well their biofilm forms are efficiently photoinactivated in the presence of light and several PSs [54,68,69,70]. The results attained with FORM in PBS when compared to its constituents, Tri-Py(+)-Me and Tetra-Py(+)-Me (Figure 2), encouraged further use to extend its benefits in the inactivation of *C. albicans* in blood plasma and/or whole blood. The results achieved with Tri-Py(+)-Me in PBS were also significant, since after 60 min of irradiation a decrease of 3.2 log_10_ (*p* < 0.05) in the fungus survival was achieved and after 270 min the detection limit was reached. As far as we are aware, this was the first time that this porphyrin derivative was used in the inactivation of *C. albicans* in plasma. Since Tetra-Py(+)-Me was the less efficient porphyrinic PS in the inactivation of *C. albicans* in PBS, it was not included in the following studies.

It is remarkable the efficiency of the porphyrinic PSs in the photoinactivation of *C. albicans* when compared to the efficiency of the reference MB, already approved for disinfection of blood plasma. In fact, MB was reported as efficient PS in the inactivation of this fungus, however, this was in concentrations 10× higher than those used in this study [70,71]. Moreover, the efficiency of MB in the inactivation of the fungus is highly dependent on the pH of the solution and the phototoxic effects only occur in the presence of saline solutions (non-buffered medium) [70].

The PBS studies give important information on the photoinactivation profile and effectiveness of the PSs, but considering the technological extension of the aPDT approach, it is also important to evaluate the efficiency of the PSs in different matrices and to compare the results with the ones obtained in buffer solutions. In this study, the evaluation of the efficiency of FORM, Tri-Py(+)-Me and MB in blood plasma and whole blood was crucial to assess the possible translation to the clinic environment. Keeping in mind that blood elements, such as erythrocytes, must not be affected by the aPDT treatment approach, the erythrocyte osmotic fragility was assessed in order to choose the safe PSs concentrations (concentrations that did not promoted hemolysis). As was observed for Tri-Py(+)-Me [5], none of the PSs had promoted hemolysis at 5.0 and 10 μM at the isotonic conditions [72] (the nonstress condition (0.9% NaCl)), which confers safety for their potential used in disinfection in whole blood and /or plasma. Although hemolysis tends to increase with the presence of reactive oxygen species (ROS) resulting from PS activation [5], this was not observed in the conditions studied, since no significant differences were observed before and after the aPDT treatment. Moreover, the fact that similar the results were attained in the dark controls and after PS irradiation, which led us to conclude that the irradiation protocol did not induce hemolysis.

At 5.0 and 10 μM concentrations of FORM, as well for Tri-Py(+)-Me, hemolysis was observed for the lowest NaCl concentrations (Figure 3). These lower NaCl concentration solutions are stress-inducing solutions, since the salt concentration in the extracellular medium is lower (hypotonic condition) than within the cell (hypertonic condition). The water enters the cell by osmosis, causing its lysis. The same hemolysis profile was achieved for the light controls (LC-Whole blood), showing, once again, that the aPDT protocol is not responsible for the red cells hemolysis. Comparing the results of porphyrinic PSs with the ones achieved for MB, were no hemolysis was observed for 5.0 μM for the 0.5, 0.7 and 0.9% of NaCl solution and for the highest concentrations of the PSs hemolysis was only achieved for the 0.3% NaCl solution (Figure 4), led us to consider that DMSO used to dissolve the porphyrinic PS has an important contribution for the hemolysis profile observed in FORM and Tri-Py(+)-Me. In fact, when the erythrocyte osmotic fragility was evaluated with 1%, 2%, and 4% of DMSO (Figure 5) the profile of hemolysis was similar to ones achieved for FORM and Tri-Py(+)-Me at 5.0, 10, and 20 μM; thus lower percentages of DMSO (1% and 2%) did not promote significant hemolysis in the 0.7% and 0.9% NaCl solutions before and after the aPDT protocol. However, in the solutions with lower percentages of NaCl, the hemolysis rates increased. For example, comparing the case of erythrocyte osmotic fragility results obtained with FORM at 5.0 μM with the ones attained with 1% of DMSO in the 0.5% NaCl solution, it was possible to observe that after the aPDT protocol, FORM induced 38% of hemolysis while DMSO endorsed 14%, which means that in fact, FORM only promoted 24% of hemolysis. This is more evident after the aPDT protocol, when FORM promoted 51% of hemolysis and DMSO endorsed 40%. DMSO is widely used in in vitro assays as a solvent of antibacterial agents and its ability to cross cell membranes is known to have an important biological feature. Regardless, with the percentages used in this work, DMSO has no effects in the survival of pathogens, including *C. albicans*. However, the hemolysis promoted by this solvent may cause some controversy regarding the use of DMSO as a solvent of PSs for blood plasma and whole blood disinfection mediated by aPDT. In this case, the choice of other drug-delivery systems such as micelles, liposomes, or the immobilization of the PS in a support may be a more secure option [73]. Nevertheless, it is important to emphasize, as already mentioned, that neither FORM or Tri-Py(+)-Me promoted hemolysis at 5.0 and 10 μM under the isotonic conditions studied.

The safe concentrations obtained for FORM and Tri-Py(+)-Me were used to photoinactivate *C. albicans* in blood plasma and whole blood. These two PSs efficiently inactivate this fungus in blood plasma, promoting a decrease in the survival of the fungus higher than the reference MB (Figure 6). In this case, Tri-Py(+)-Me seems to be the more efficient PS on the inactivation of *C. albicans* in blood plasma, since the decrease attained in the survival of the fungus after 270 min of irradiation was higher than the one observed for FORM (2.5 log_10_ *vs* 1.7 log_10_ for 10 μM of each PS). This was quite surprising, since in the PBS assays FORM was the most efficient PS in photoinactivation of *C. albicans*. This may be explained, not only by the complexity of the plasma matrix, which is rich in several proteins that can interfere by trapping the PS, but also due to the complexity of FORM. This formulation is constituted by 5 cationic porphyrins with different number of charges. The constituents of this formulation can, in a complex environment such as plasma, lose their ability to efficiently bind to the microbial membrane cells, decreasing their photodynamic efficiency. In regards to the reference MB, this was the less efficient PS in the inactivation of the fungus (promoting a decrease of *c.a* 0.5 log_10_ for the higher tested concentration), maintaining the inactivation profile at a constant level between 90 and 270 min. The reduced efficiency of MB can be explained by the fact that this PS has only a positive charge. It is well known that cationic PS with 3 or 4 charges are more effective for photoinactivating microorganisms, namely Gram negative bacteria and fungi, than neutral PS or PS with only one or two positive charges [46,74]. The observed photodegradation of MB can also, at least in part, justify the poor ability of these PS in the photoinactivation of *C. albicans* in blood plasma. Moreover, MB at 5.0 and 10 μM was not more efficient than FORM and Tri-Py(+)-Me at the same concentrations, so it is expected that, at the same amounts used in the approved methodology for plasma disinfection mediated by light and MB (where the PS is used at 0.8–1.2 μM), the porphyrinic PSs continued to be more efficient than MB [75].

While it is known that the PSs approved to disinfect plasma are not approved for disinfecting concentrated erythrocytes and platelets due to the negative effects observed in these elements after aPDT, the ability of FORM and Tri-Py(+)-Me to photoinactivate *C. albicans* in whole blood was still studied (Figure 7). However, under the aPDT protocol selected, the decrease of the fungus survival attained 0.7 log_10_ for the higher concentrations of each PSs. Once again, the complexity of the blood matrix may be the answer for this limited efficiency of the PSs. The nonspecific binding of the PS to blood proteins and to the high number of elements coating cell membranes can decrease the efficiency of the photoinactivation [5]. Moreover, while the microorganisms are in the suspension in plasma, in the whole blood pathogens may be in suspension or associated with cells (intracellular or extracellular), which can undermine the interaction of the ROS with the membrane of the cells [7]. Nevertheless the potential application of FORM and Tri-Py(+)-Me in the photoinactivation of *C. albicans* in blood plasma is very promising.

In order to investigate whether treated plasma mediated by aPDT could damage the erythrocyte membranes after the transfusion, the erythrocyte osmotic fragility after aPDT of plasma with FORM, Tri-Py-(+)-Me at concentrations of 5.0 and 10 μM was studied. The results were also compared to the ones achieved for MB at the same concentrations. This study aims to simulate plasma transfusions and to evaluate the possible erythrocyte damage that aPDT-treated plasma transfusion can cause in the receptor. None of the PSs had promoted hemolysis at 5.0 and 10 μM at the isotonic conditions (0.9% NaCl) before aPDT and after aPDT, followed by dark incubations of 30 min and 6 h. These results confirm the safety of the use of FORM and Tri-Py-(+)-Me in the disinfection of plasma, after a transfusion.

## 4. Materials and Methods

### 4.1. Blood Samples

Human blood samples were voluntarily provided by Avelab clinical laboratory (Aveiro, Portugal). The blood tubes provided contains whole blood but also 5.4 mg of an anticoagulant-EDTAk_3_ prefacing a final volume of 3 mL (BD Vacutainer^®^, Becton Dickinson, Plymouth, UK). The blood samples were used for up to 5 days after being received and were stored under appropriate conditions. Plasma was obtained after centrifugation of whole blood at 3500 rpm (Heraeus Megafuge 16R, Waltham, MA, USA) for 5 min.

Fresh human blood samples (<24 h) used for erythrocyte osmotic fragility assays were kindly provided by volunteers and collected at CMM- Aveiro Medical Center. The blood tubes contain 16.2 mg of EDTAk_3_ prefacing a final volume of 9 mL (Vacumed^®^, Torreglia, Italy).

### 4.2. Characterization of Microbial Strains and Culture Conditions

The yeast *C. albicans* (ATCC 10231) was maintained on Yeast Extract Glucose Chloramphenicol Agar (YGCA, Liofilchem, Roseto degli Abruzzi, Italy) at 4 °C. Before each assay, a colony was transferred to 20 mL of YG [Yeast extract (5 g/L) + Glucose (10 g/L)] and incubated for 24 h at 37 °C with constant stirring (120 rpm). Then 200 μL aliquots were transferred to new 20 mL YG and incubated at the previous growth conditions in order to reach the stationary phase, corresponding to a concentration of 10^6^–10^7^ colony forming units per mL (CFU·mL^−1^).

### 4.3. Light Sources

The efficiency of the PSs in PBS was evaluated by exposing the samples and controls of a set of 13 white fluorescent lamps (PAR radiation, OSRAM 21 lamps of 18 W each, 380–700 nm) for a maximum irradiation period of 270 min with an irradiance of 2.5 mW·cm^−2^.

In aPDT assays for plasma and whole blood, the samples and controls were irradiated with a compatible fiber optic probe attached to a 250 W quartz/halogen lamp (LUMACARE model 122, Newport Beach, CA, USA) with an irradiance of 150 mW·cm^−2^ for a maximum irradiation period of 270 min. All the irradiations were measured with a Coherent FieldMaxII-Top combined energy meter (COHERENT, Santa Clara, CA, USA).

### 4.4. Photosensitizers

Stock solutions of FORM and Tri-Py(+)-Me were prepared in DMSO at a concentration of 500 μM and stored in the dark. Stock solution of MB was prepared in PBS at 500 μM and stored in the dark. All photosensitizers were sonicated for 30 min before each assay (ultrasonic cleaner, Nahita 0.6 L, 40 kHz, GT SONIC Technology, Guangdong, China).

The porphyrins 5,10,15-tris(1-methylpyridinium-4-yl)-20-(pentafluorophenyl)porphyrin tri-iodide (Tri-Py(+)-Me), 5,10,15,20-tetrakis(1-methylpyridinium-4-yl)porphyrin tetra-iodide (Tetra-Py(+)-Me) and the formulation (FORM) [a mixture of non-separated porphyrins: 5-(1-methylpyridinium-4-yl)-10,15,20-tris(pentafluorophenyl)-porphyrin mono-iodide (Mono-Py(+)-Me) (19%), 5,15-bis(1-methylpyridinium-4-yl)-10,20-bis(pentafluorophenyl)porphyrin di-iodide (Di-Py(+)-Me *opp*) and 10-bis(1-methylpyridinium-4-yl)-15,20-bis(pentafluoro- phenyl)porphyrin di-iodide (Di-Py(+)-Me *adj*) (20%), Tri-Py(+)-Me (44%) and Tetra-Py(+)-Me (17%)] were synthetized according with the literature and their structures are presented in Figure 1 [65,66,74]. The UV-Vis spectrum of these photosensitizers was already reported in the literature [64,65].

### 4.5. Antimicrobial Photodynamic Assays

The *C. albicans* culture, after reaching the stationary phase was diluted (1:10) in the selected matrix: PBS, plasma, and whole blood. Then, the resulting suspension was then distributed to the wells of a 12-well plate. Plasma was obtained by centrifugation of whole blood at 3500 rpm for 5 min (Heraeus Megafuge 16R, Waltham, MA, USA). Each PS was then added to the samples making a final concentration of 5.0 μM in PBS, 5.0 μM and 10 μM in plasma, and finally 10 μM in whole blood. Light and dark controls were also carried out simultaneously with the aPDT procedure: the light controls (LC) comprised a *C. albicans* suspension and the dark control (DC) comprised the fungus suspension incubated with the PSs at the higher concentration tested protected from light. To promote the interaction of the photosensitizer with the fungus, samples and controls were incubated before irradiation for 10 min in PBS and 30 min in plasma and whole blood under constant agitation in the dark. Increased incubation time for plasma and whole blood was required due to the protein and cellular complexity of these matrices, which made the interaction more challenging.

Samples and light controls (LC) were irradiated under the conditions described above while the dark control was protected from light during treatment. The samples and each control were kept in agitation at a controlled temperature of 25 °C. The photoinactivation capacity of each PS was evaluated by quantifying the number of CFU per volume (CFU·ml^−1^). Aliquots of samples and of each control were taken at time 0 min (after incubation time) and at different irradiation times (15, 30, 60, 90, 180, and 270 min). After this, serial dilutions were made and finally plated in YGCA and incubated for 4 days at 37 °C. The same conditions were used in all experiments, for each condition three independent assays with two replicates each were performed.

### 4.6. Evaluation of aPDT and DMSO Effect on Erythrocyte Osmotic Fragility

The effect of aPDT on erythrocyte osmotic fragility was evaluated using 5.0, 10, and 20 μM of FORM and MB. These assays were already described in the literature [5,76]. The erythrocyte osmotic fragility was evaluated before (incubation time) and after aPDT treatment; firstly, the PS was added to samples and dark controls, and the resulting samples and controls were incubated for 30 min in the dark. Thus, samples and light controls were irradiated at 150 mW·cm^−2^. Aliquots of 20 μL of each samples and controls were added to eppendorf tubes with 1980 μL of NaCl solutions (0, 0.1%, 0.3%, 0.5%, 0.7%, 0.9%) and were incubated at 25 °C for 30 min with constant stirring. Finally, all samples were centrifuged at 3500 rpm for 10 min (Gyrozen 1730R, Gimpo, Korea), following which the supernatants resulting from this centrifugation were collected and their optical densities were measured. The optical density of the supernatant was determined spectrophotometrically (Multiskan FC, Thermo Scientific, Waltham, MA, USA) at 540 nm, the wavelength recommended for evaluating the amount of hemoglobin in solution. Hemolysis was represented in percentage by considering the optical density value of distilled water solution (0% NaCl) as 100%. A similar procedure was performed but using different percentages of DMSO (1%, 2%, 3%, and 4%).

### 4.7. Evaluation of Erythrocyte Osmotic Fragility after the Addition of the Treated Plasma with FORM, Tri(+)-Py-Me, and MB to the Concentrated Erythrocytes

**Figure 11:**
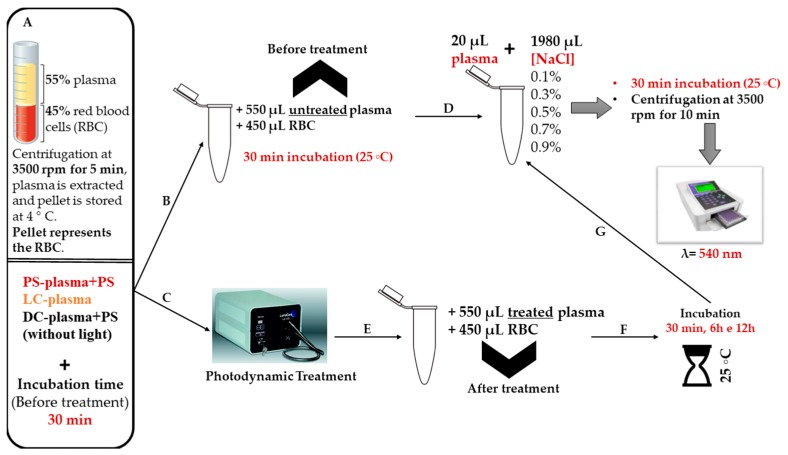
Schematic representation of the experimental protocol to evaluate the erythrocyte osmotic fragility after the addition of the treated plasma with FORM, Tri-Py(+)-Me, and MB to the blood components.

These assays were performed in order to evaluated possible negative effects in erythrocyte membranes that aPDT-treated plasma transfusion with FORM, Tri-Py(+)-Me, and MB can cause in the receptor. For this purpose, the erythrocyte osmotic fragility induced by untreated (after incubation time) and treated (after aPDT) plasma was evaluated. Initially the whole blood was centrifuged at 3500 rpm for 5 min (Heraeus Megafuge 16R, Waltham, MA, USA) to extract the plasma, while the centrifugation pellet containing the red blood cells was stored at 4 °C. After collecting the plasma (for treatment), the PS was added to samples and dark controls, and both samples and controls were incubated for 30 min in the dark. Then, the samples and light controls were irradiated (150 mW·cm^−2^) and the dark controls kept in the dark. The treated plasma was incubated with the red blood cells concentrates for 30 min and 6 h and so the effect of treated plasma on red blood cells at short and long term was evaluated. For this, the interaction of treated plasma and the red blood cells concentrates was prepared in eppendorf tubes at a final volume of 1 mL in a proportion of 55% plasma/45% red blood cells [3]. Each eppendorf tube was incubated at 25 °C for 30 min and 6 h with constant stirring. All of the remaining steps of the protocol were the same as the ones performed in the previous section (Figure 11).

### 4.8. Statistical

At least three independent experiments with two replicates per assay for each condition were done. The statistical analysis was performed with GraphPad Prism (GraphPad Software, San Diego, CA, USA). Normal distributions were checked by the Kolmogorov–Smirnov test and the homogeneity of variance was verified with the Brown Forsythe test. ANOVA and Dunnet’s multiple comparison tests were applied to assess the significance of the differences between the tested conditions. A value of *p* < 0.05 was considered significant.

## 5. Conclusions

It is obvious that there is a lack of efficient methods for the inactivation of pathogens in blood plasma and whole blood [5,7,18], and it is crucial to develop new strategies to inactivate microorganism in plasma and/or blood [10]. aPDT can represent an alternative to the conventional disinfection techniques since it is a non-antibiotic approach that has been shown to be effective in the photoinactivation of several pathogens. One of the disinfection techniques approved for pathogenic inactivation in plasma considered the use of MB as PS in an aPDT approach. This work shows that the porphyrinic formulation FORM and the Tri-Py(+)-Me are promising PSs in the inactivation of *C. albicans* in blood plasma, causing higher inactivation rates than MB. Moreover, these porphyrinic PSs had shown no significant negative effects on the erythrocytes in isotonic conditions when hemolysis was evaluated in whole blood and after the addition of treated plasma to the concentrated blood cells. However, further studies are needed to overcome the barriers that the complex matrix of whole blood promotes in order to improve the efficacy of FORM and Tri-Py(+)-Me in the photoinactivation of *C. albicans* in whole blood.
